# Supplementary material for: Stylometry can reveal artificial intelligence authorship, but humans struggle: A comparison of human and seven large language models in Japanese
Source: PLoS One. 2025 Oct 27;20(10):e0335369. doi: 10.1371/journal.pone.0335369 (PMC12558491; doi:10.1371/journal.pone.0335369)
Supplement: Attachment — Submitted filename: Response to Reviewers.docx [file pone.0335369.s006.docx]

Answer and Response to **Reviewer 1** comments:

Thank you for your constructive review.

I revised several points along with your comments.

Please confirm revised paper.

Thank you.

Major Comments

1. Scope and Generalizability

We added limitations in final of general discussion along with your comment as follows:

“We obtained the above results, but the next main limitations should be noted. (1) In both studies, we analyzed only Japanese public comments. However, writing style in Japanese is likely influenced by sentence formality (formal vs. informal), constraints imposed by format (e.g., academic papers), and genre (e.g., creative writing, essays, social media, email). Therefore, the above results may limit generalizability, it is necessary to target a wider range of media to confirm this study’s conclusions.”

2. Classification Methodology:

Concerned this points, we think it is enough to demonstrate clear different distribution between LLMs and Human by using MDS. So RF classifier is just supplementary analysis because it is natural that they can be neatly classified.

But for reference we conducted sensitivity analysis as follows:

“In addition, sensitivity analyses, tuned across each hyperparameter (ntree, mtry, maxnodes, and nodesize) together with down-sampling to balance classes from AI=350/Human=100 to AI=100/Human=100, also showed stable performances.”

3.Study 2 Design Limitation:

We added limitations in final of general discussion along with your comment as follows:

“(2) In Study 2, the experimenters presented only one text in each LLM to participants. Texts generated by same LLMs were different in terms of content, wording, and phrasing in addition to writing style as Fig 1–4 showed distributions. We attempted to control only writing-style biases within same LLMs by including distance differences into the Bayesian model, but we did not control the other factors. Future studies should present multiple texts per LLM and evaluate content effects to improve generalizability.”

4. Connection to Prior Work

We revised paragraph before Table2.

“In summary, the current study demonstrated unreliable accuracy levels of the participants’ AI detection in line with previous reports of chance-level human performance [5-6].”

5. Interpretation of Stylometric Differences:

We revised this point by only adding one word “hypothesis”.

“(l.458) Therefore, it is possible hypothesis that the number of parameters is related to distinct stylometric features.”

6. Presentation of Results:

In this point, we have already intended to write in each key point (e.g., The odds rate (1.40(=exp(0.34)) for GPT-o1 indicates that the participants had stronger confidences in their own decisions compared to with GPT-4o). OK?

Minor Comments

Language and Typos:

We revised sentences along with your comments.

By the way, we had already a native person check the manuscript.

Clarity of Terms:

We revised as “GPT-4o” and “GPT-o1”.

Figure Captions:

We added captions as follows:

Fig 1. MDS configuration for function word unigrams in eight classes including each LLM and human.

* The distances (e.g., symmetric Jensen–Shannon distances) between each object on the two dimensions indicate relative similarity of concerned stylometric features.

Data Availability:

We will reposit our dataset on PLOS ONE site and intend to write that down in the input form.

Ethics Statement:

We moved “ethics statements and informed consent” into Methods along with your comment.

Reference to Self-Published Work:

Along with your comment, we added the following sentence in introduction.

“In belief, this study extended these previous studies by evaluating new text (e.g., public comments) using multiple LLMs and by examining a human-judgment as stated next.”

Answer and Response to **Reviewer 2** comments:

Thank you for your constructive review.

I revised several points along with your comments.

Please confirm revised paper.

Thank you.

1. Study 2 Design Limitation:

We added limitations in final of general discussion along with your comment as follows:

“(2) In Study 2, the experimenters presented only one text in each LLM to participants. Texts generated by same LLMs were different in terms of content, wording, and phrasing in addition to writing style as Fig 1–4 showed distributions. We attempted to control only writing-style biases within same LLMs by including distance differences into the Bayesian model, but we did not control the other factors. Future studies should present multiple texts per LLM and evaluate content effects to improve generalizability.”

2. Language and Typos:

We revised sentences along with your comments.

By the way, we had already a native person check the manuscript.

3. MDS and violin plots

We added captions of MDS as follows:

Fig 1. MDS configuration for function word unigrams in eight classes including each LLM and human.

* The distances (e.g., symmetric Jensen–Shannon distances) between each object on the two dimensions indicate relative similarity of concerned stylometric features.

But caption of violin plots have been written as follows.

“Fig 5. Violin plots and box-and-whisker plots of judgments of participants (a 7-point Likert scale) against texts corresponding to each LLM and human

* Each violin plot with light blue shows the shape of the distribution via a kernel density estimate. The red diamond in a box-and-whisker plot represents the median of the distribution.”

4. The finding for ChatGPT-o1

We revised along with your comment as follows in abstract.

“Moreover, in our materials, more advanced ChatGPT(o1), plausibly reflecting relatively greater fluency and polish, tends to mislead the participants to believe “human-written” texts compared with ChatGPT(GPT-4o) and improves their confidence for their own judgments.”

Answer and Response to **Reviewer 3** comments:

Thank you for your constructive review.

I revised several points along with other reviewers’ comments.

Please confirm revised paper.

Thank you.

Answer and Response to **Reviewer 4** comments:

Thank you for your constructive review.

I revised several points along with your comments.

Please confirm revised paper.

Thank you.

1. Restrict of writing styles

We focused on only three features (phrase patterns, POS bigrams, and function words unigrams) because we expected them to provide sufficient discriminatory power. As we expected, our results confirmed this. Exploring higher-level features is valuable but was outside the scope of the present study and is not required for our current conclusions.

2. Standardized distances

We have already written this method of standardized distances in Supporting information or footnote. But we made some modifications as follows.

“*1 Because the self-distance between human-written texts is naturally zero, this study standardized distance as follows: First, we calculated the distances between the target human-written text (presented in Fig 4) and the other 99 human-written texts and computed the mean of these distances (i.e., within-group mean). The original distance value for the human stimulus (e.g., zero) was replaced with the average value. Next, we standardized all distance values based on the mean and standard deviation calculated from the distances between the replaced target human-written text and the other texts (i.e., both LLM-generated and the remaining 99 human-written texts).”

3. The other methods

As explained in the previous article, we did not use t-SNE because of reproducibility with random initialization. Moreover, MDS is considered as valid method compared with other statistical method (e.g., PCA) for Japanese authorship verification. Furthermore, we think that given that our metric-MDS map already cleanly separates the classes and it is not necessary to use further alternative methods.

4. Random Forest:

Concerned this point, we think it is enough to demonstrate clear different distribution between LLMs and Human by using MDS. So RF classifier is just supplementary analysis because it is natural that they can be neatly classified.

But for reference we conducted sensitivity analysis as follows:

“In addition, sensitivity analyses, tuned across each hyperparameter (ntree, mtry, maxnodes, and nodesize) together with down-sampling to balance classes from AI=350/Human=100 to AI=100/Human=100, also showed stable performances.”

5. Text genre and Generalizability

We added limitations in final of general discussion along with your comment as follows:

“We obtained the above results, but the next main limitations should be noted. (1) In both studies, we analyzed only Japanese public comments. However, writing style in Japanese is likely influenced by sentence formality (formal vs. informal), constraints imposed by format (e.g., academic papers), and genre (e.g., creative writing, essays, social media, email). Therefore, the above results may limit generalizability, it is necessary to target a wider range of media to confirm this study’s conclusions.”

6. Zero-shot prompting:

We added limitations in final of general discussion along with your comment as follows:

“(3) This study limited only texts generated via zero-shot prompting. As stated in the Introduction, prior work [11] reports that texts generated by one-shot prompting are more challenging to analyze compared with ones via zero-shot prompting. In future work, it will be necessary to examine the distributions of texts generated by one- or few-shot prompting.”
